# Biocontrol of bacterial seedling rot of rice plants using combination of *Cytobacillus firmus* JBRS159 and silicon

**DOI:** 10.1371/journal.pone.0290049

**Published:** 2023-08-14

**Authors:** Jun An Kang, Swarnalee Dutta, Yong Hoon Lee

**Affiliations:** 1 Division of Biotechnology, Jeonbuk National University, Iksan-si, Jeollabuk-do, Republic of Korea; 2 Advanced Institute of Environment and Bioscience, Plant Medical Research Center, and Institute of Bio-industry, Jeonbuk National University, Jeonju-si, Jeollabuk-do, Republic of Korea; University of Agriculture Faisalabad, PAKISTAN

## Abstract

*Burkholderia glumae* causes bacterial panicle blight (BPB) and bacterial seedling rot (BSR) which are difficult to control in rice plants. Seed disinfection using microbes and eco-friendly materials is an efficient alternative practice for managing BPB and BSR. In this study, we applied *Cytobacillus firmus* JBRS159 (JBRS159) in combination with silicon dioxide (SiO_2_) nanoparticle or potassium silicate (K_2_SiO_3_) solution to control BSR. JBRS159, SiO_2_ nanoparticle, and K_2_SiO_3_ independently suppressed the BSR disease and promoted growths of rice and Arabidopsis. Population of *B*. *glumae* in the treated rice seeds was suppressed by the application of JBRS159 via competitions for nutrients and niches. The mixture of JBRS159 and each Si compound (SiO_2_ nanoparticle or K_2_SiO_3_) was complementary for disease-suppressing and growth-promoting activities of individual treatment. The results of this study indicate that mixture of JBRS159 with each Si compound can be harnessed for disease control and growth promotion as efficient alternatives to chemical pesticides and synthetic fertilizers. The efficacy of JBRS159 and Si compounds in the control of BSR and BPB in the field remains to be evaluated.

## Introduction

Bacterial panicle blight (BPB) caused by *Burkholderia glumae* has become a serious problem in major rice-growing countries [1–4] and is predicted to occur more frequently and severely because of high temperatures and frequent rainfall during the rice-growing season [5,6]. *Burkholderia glumae*, a seed-borne pathogen, also causes bacterial seedling rot (BSR) as well as sterility of spikelets and reduction of grain weight [7,8]. As chemical control of the diseases is not effective and completely resistant cultivars are not available [9], BPB and BSR result in serious quality and yield losses. Therefore, effective strategies should be developed for disease management during the germination and flowering stages of rice plants.

Biological control using microorganisms has been used to control *B*. *glumae* either individually or as part of an integrated management program. Pretreatment of rice seeds with avirulent strains of *B*. *glumae* significantly suppressed the incidence of BSR caused by virulent strains [10]. An avirulent *Burkholderia gladioli* strain also prevented the occurrence of the disease when rice panicles were co-inoculated with virulent *B*. *glumae* [11]. *Pseudomonas protegens* PBL3 having antimicrobial activity against *B*. *glumae in vitro* and *in planta* reduced disease symptoms under greenhouse conditions [9]. *Bacillus* species, including *Bacillus velezensis* IBUN2755, putatively reduced the endophytic population of *B*. *glumae* in both the roots and shoots of rice plants by niche competition, reducing the presence of empty grains [12,13]. The use of *B*. *glumae*-contaminated seeds and transplantation of young seedlings after cultivation in nursery boxes promotes bacterial populations in plants and causes epidemics in rice nurseries and fields [14]. Therefore, the use of pathogen-free seeds is recommended to reduce disease incidence. The introduction of microorganisms possessing plant growth-promoting or biocontrol activities into or on seeds is a promising approach for increasing crop yield and health [15]. For instance, biopriming rice seeds with potential bacterial strains improves growth and suppresses bacterial blight [16].

Despite many successful applications of biocontrol agents, their use as biocontrol products is limited because of their inconsistent performance in field applications [17]. A combination of microbial biocontrol agents and other chemical components has been reported to increase biocontrol efficacy. For instance, many salt additives, such as calcium chloride, sodium carbonate, and sodium bicarbonate, along with microbial biocontrol agents efficiently controlled diseases in fruits and vegetables [18,19]. Silicon, the second most abundant element (27%) in the earth’s crust, is usually present in unavailable forms of silicates that cannot be readily used by plants [20]. Although several plants do not require Si, Si is absorbed as soluble monosilicic acid, resulting in the strengthening of the cell wall, which increases resistance to various abiotic and biotic stresses in many crops, including rice and wheat [21–26]. Rice takes up high amounts of Si, varying from 0.1% to 10% dry weight of shoots, making the cell walls thick and rigid, and in-turn, increasing resistance to pathogen penetration and lodging [20]. Silicon nanoparticles are an effective alternative to Si as part of conventional mineral fertilizers [23,27]. Silicon (di)oxide nanoparticles have been suggested to increase cell wall thickness and enhance resistance to pathogen penetration and drought stress [28,29]. El-Shetehy et al. [30] reported that SiO_2_ nanoparticles and soluble orthosilicic acid (Si(OH)_4_) induced systemically acquired resistance via salicylic acid signaling. The authors concluded that SiO_2_ nanoparticles have the potential to be used as an inexpensive, highly efficient, safe, and sustainable alternative to control plant diseases.

In the present study, to control BSR caused by *B*. *glumae*, we isolated a potential bacterial strain, *Cytobacillus firmus* JBRS159 (JBRS159), from the seeds of rice plants and assessed its biocontrol activity against BSR. To increase biocontrol effectiveness, JBRS159 was applied together with silicon dioxide (SiO_2_) nanoparticles or soluble potassium silicate (K_2_SiO_3_) solution, and the underlying mechanisms were investigated. The effects of JBRS159 and Si compound (K_2_SiO_3,_ and SiO_2_ nanoparticle) on plant growth promotion were also explored. Our results showed that JBRS159 and Si compounds are useful for protection of rice plants from BSR and growth promotion of rice plants.

## Materials and methods

### Isolation and identification of JBRS159

Endophytic bacteria were isolated from rice seeds as previously described by Dutta et al. [31]. Briefly, surface sterilized rice seeds were macerated in a sterilized mortar and pestle. The homogenates serially diluted and plated onto 1/10 trypic soy agar (TSA) medium containing 1% rice seed exudates, and then incubated at 30°C for 3 d. The bacterial colonies were picked up and transferred to a 1.5 mL tube containing Luria-Bertani (LB) medium with 15% (v/v) glycerol. The tubes were incubated at 30°C for 24 h and stored at −80°C until further biological analysis. The potential biocontrol agent JBRS159 identified as *Cytobacillus firmus* was selected and used for this study. The entire genome of JBRS159 was sequenced and deposited in the National Center for Biotechnology Information database (Acc. No. JAQZDS000000000) [32].

### Preparation of *B*. *glumae*-infected rice seeds

Artificially infected rice seeds were prepared as described by [33], with minor modifications. Healthy rice (‘Sukwang’) seeds, widely cultivated in the southern part of South Korea, were surface sterilized with 2% sodium hypochlorite for 2 min and then washed with sterile distilled water (sDW). A bacterial suspension of *B*. *glumae* was cultured in LB broth medium for 24 h at 28°C and 180 rpm and adjusted to 1 × 10^8^ cfu mL^-1^ in sDW supplemented with 0.2% carboxymethyl cellulose (CMC). The surface-sterilized rice seeds were soaked in the *B*. *glumae* suspension (10 g seeds 100 mL^-1^) for 12 h at 25°C and 100 rpm. The challenged seeds were air-dried for 12 h at room temperature and used as challenged seeds for biocontrol assays.

### Application of JBRS159 and Si compound to control BSR

The strain JBRS159 was grown in LB broth at 28°C for 24 h and the bacterial cells were adjusted to 1 × 10^6^, 10^7^, and 10^8^ cfu mL^-1^ in sDW amended with 0.2% CMC. The *B*. *glumae*-inoculated rice seeds were immersed in each concentration of JBRS159 suspension for 1 h at 28°C and 100 rpm to facilitate the attachment of bacterial cells to the seed coat. Soluble silicate in the form of K_2_SiO_3_ solution (Samchun Chemicals, Seoul, South Korea) and SiO_2_ nanoparticles (<50 nm; Sigma-Aldrich, USA) were used in this study. To determine the optimum concentration for Si application, infected seeds were soaked in different concentrations (50, 100, 200, and 400 mg L^-1^) of K_2_SiO_3_ suspensions or SiO_2_ nanoparticles for 4 h at room temperature and 100 rpm (5 g seeds per 20 mL suspension). The seeds were placed on sterilized filter paper to remove excess liquid and air-dried for 15–20 min. For combination treatment, *B*. *glumae*–infected rice seeds were soaked in a bacterial cell suspension supplemented with optimum concentration of K_2_SiO_3_ or SiO_2_ nanoparticles (100 mg L^-1^). The treated seeds were sown in pots filled with the nursery bed soil (Pungnong Co., Korea) used for the cultivation of rice seedlings. Seedlings were raised in a plant growth room (16/8 h, light/dark, 28°C), watered daily, and did not receive additional fertilization. Seeds treated with sDW amended with CMC served as control. A prochloraz-copper chloride-tebuconazole (Hankooksamgong Co., South Korea) suspension, which is recommended for the control of rice grain rot, was used according to the manufacturer’s instructions as a chemical control. Each treatment consisted of three replicates of 100 seeds each and the experiment was repeated thrice. At 21 days after sowing, seedlings were rated on a 0–4 scale for the disease severity where 0 = seedlings with no symptoms and vigorous as control, 1 = seedlings with pale yellow leaves, 2 = seedlings with severe chlorosis and stunting, 3 = seedlings with complete discoloration and rotting, and 4 = seeds completely rotted without development (S1 Fig) [34]. Disease index on a percentage basis was calculated as [sum (class frequency × score of rating class)] / [(total number of plants) × (maximal disease index)] × 100.

### Plant growth promotion assay of JBRS159 and Si compounds

*Arabidopsis thaliana* ecotype Columbia-0 (Col-0) seeds were surface sterilized with 70% (v/v) ethanol for 90 s and 1% (v/v) sodium hypochlorite for 5 min, and then washed thrice with sDW. The disinfected seeds were soaked in a JBRS159 suspension supplemented with K_2_SiO_3_ or SiO_2_ nanoparticles (100 mg L^-1^) at 28°C in a rotary shaker at 150 rpm for 30 min. The excess moisture was removed using sterilized filter papers, and the seeds were placed in Petri dishes (90 × 15 mm) containing half-strength Murashige and Skoog (1/2 MS) medium supplemented with 1.5% sucrose and 0.8% (w/v) agar. The plates were sealed with parafilm and placed at an angle of 70° in plant growth chambers under light cycle (16/8 h light/dark; 100 µmol m^-2^ s^-1^) conditions at 23 ± 1°C. The seeds soaked with sDW amended with 0.2% CMC served as controls. After 14 d of incubation, the number of lateral roots of individual seedlings and the fresh weight of the plants were measured. The experiment consisted of three replicates of five seeds each and the entire experiment was repeated thrice. Rice seeds challenged with *B*. *glumae* and treated with a JBRS159 suspension supplemented with each Si compound were also analyzed for growth promotion. The fresh and dry weights of rice plants were recorded 21 d after sowing and compared with those of plants challenged with the pathogen alone.

### Antagonistic activity assay of JBRS159 and Si compounds

The antagonistic activity of JBRS159 against the bacterial pathogens *B*. *glumae*, *B*. *plantari*, and *B*. *gladioli* (bacterial grain rot of rice), *Acidovorax avenae* (bacterial stripe), and *Xanthomonas oryzae* pv. *oryzae* (bacterial leaf blight) was tested using overlay inoculation [35]. Cells of JBRS159 that were grown for 24 h was adjusted to 1 × 10^7^ cfu mL^-1^ and spot inoculated (20 μL) on paper disks (8 mm) that are laid on the test plates. Inhibition zones were measured 2 d after incubation at 28°C. The experiment was replicated twice with three plates per replicate.

The antagonism of each Si compound against pathogen *B*. *glumae*, biocontrol agent JBRS159, and beneficial bacterial strains such as *Bacillus velezensis*, *Burkholderia pyrrocinia*, and *Pseudomonas parafulva* JBCS1880 [31,35] that have been known to promote plant growth was tested using overlay inoculation methods. Test plates were prepared using LB agar medium mixed with cells (1 × 10^6^ cfu mL^-1^) of each bacterial strain, and a 20 µL Si suspension (100, 200, 500, and 1000 mg L^-1^) of K_2_SiO_3_ and SiO_2_ nanoparticle was inoculated on the paper disks. Plates were observed for inhibition zones 2 d after incubation and experiments were replicated twice with three plates per replicate.

### Estimation of *Burkholderia glumae* population in rice seeds

A spontaneous chloramphenicol-resistant *B*. *glumae* mutant was used for the estimation of its population. Rice seeds were challenged with the chloramphenicol-resistant *B*. *glumae* and treated with JBRS159 as described above, and then seeds were collected 0 and 1 d after sowing, and radicles were sampled 2 d and 3 d after sowing. The seeds and/or roots attached with rhizosphere soil were vortexed for 20 to 30 s in sDW. Serial 10-fold dilutions were plated on LB medium containing chloramphenicol (50 µg mL^-1^). The plates were incubated at 28°C for 48 h and cell numbers were calculated.

### Nutrient competition assay

The polytetrafluoroethylene (PTFE) membrane assay [36] was used to assess the nutrient competition between *B*. *glumae* and JBRS159 with minor modifications. Briefly, the wells of a 12-well culture plate were filled with JBRS159 suspension (1 × 10^5^ cfu mL^-1^) in nutrient-limited (10% rice seed exudate -RSE) [27] or nutrient-rich (10% LB or full-strength LB) broth (0.6 ml per well). Cylindrical inserts with PTFE membrane (pore size 0.45 μm) attached at the bottom were placed in the wells and allowed to stand for 2 min to ensure the membrane was completely moist. The chloramphenicol-resistant *B*. *glumae* suspension in sDW (0.4 ml of 1 × 10^5^ cfu mL^-1^ suspension per cylinder) was then placed in the cylinder, and the plates were incubated at 28°C. Wells containing only 10% RSE, 10% LB or full-strength (100%) LB with cylindrical inserts containing *B*. *glumae* suspension served as positive controls while wells with respective media and PTFE membrane filled with sDW served as negative controls. After 24 h of incubation, the cylinders were removed from the wells, and excess liquid from the membrane was blotted with tissue paper. The membrane was cut out with a clean scalpel and transferred to 10 ml sDW for serial dilution and plating in LB medium containing chloramphenicol (50 µg mL^-1^). The plates were incubated at 28°C for 48 h and cell numbers of *B*. *glumae* were calculated.

### Plant growth-promoting and pathogen-suppressing compound production assay

Siderophore production by JBRS159 was determined using the modified chrome azurol S agar method [31,37] and was observed as an orange halo around the colony. Hydrogen cyanide (Schwyn B, Neilands JB (1987) Universal chemical assay for theHCN) production was determined by observing the development of pink coloration [38]. Proteolytic activity was determined using skim milk agar plates [39], phosphate solubilization ability was determined using Pikovskaya agar medium [40], and silicate solubilization ability was determined using glucose media to check for a clear zone around the colony [41]. All experiments were performed in triplicate.

### Phytohormone production assay

Indole 3-acetic acid (IAA) production was determined using the Salkowski reagent [42] and measured spectrophotometrically using a SpectraMax-250 microplate reader at 536 nm and quantified using a standard curve. Cytokinin production was quantified spectrophotometrically at 665 nm [43] using M9 medium supplemented with 0.2% casamino acids, 0.01% thiamine, and 2 pg of biotin per liter [44]. To determine gibberellic acid production, an ethyl acetate extract of JBRS159 grown in nutrient medium was used. For estimation, gibberellic acid was converted to gibberellenic acid and quantified at an absorbance of 254 nm [45]. All experiments were conducted in triplicates.

### Statistical analysis

The experiments were done in completely randomized design and the data were subjected to analysis of variance using the SAS JMP software (SAS Institute, Cary, USA). Significant differences were determined from the treatment means using the least significant difference (LSD) test at *P* = 0.05. Data from each experiment were analyzed separately. The population of *B*. *glumae* in seeds and radicles was analyzed using the Student’s *t-*test at P<0.05.

## Results

### Biocontrol effects of JBRS159 against BSR

**Fig 1:**
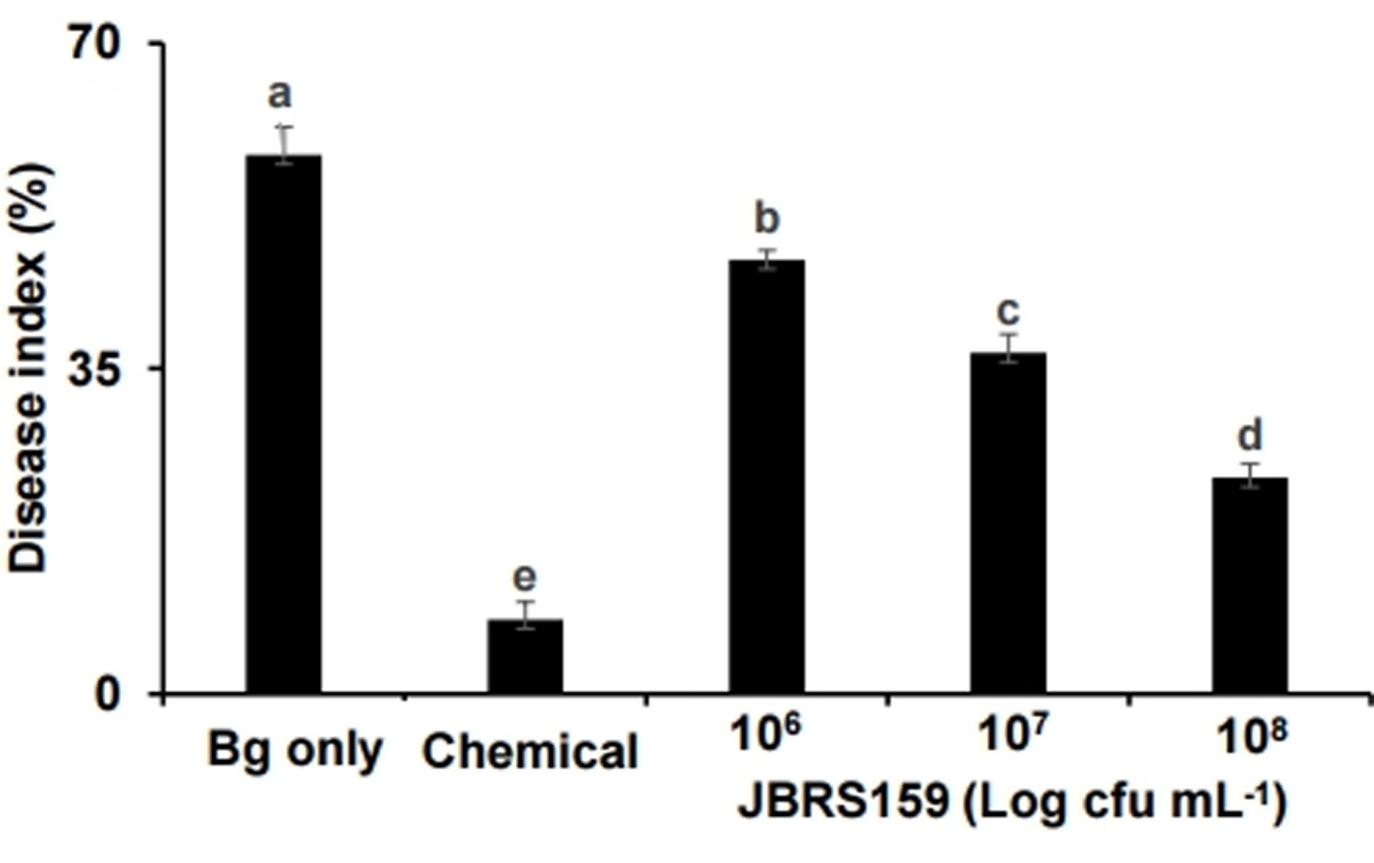
Control of bacterial seedling rot by treatment with various cell concentrations of *Cytobacillus firmus* JBRS159. Surface-sterilized rice seeds were challenge inoculated with *Burkholderia glumae*, air-dried, and soaked in 1 × 10^6−8^ cfu mL^-1^ of *C*. *firmus* JBRS159 (JBRS159) cells. The treated seeds were sown in pots containing nursery soil and disease index was calculated 21 d after inoculation. The seeds treated with sterile distilled water (DW) amended with carboxymethyl cellulose (CMC; Bg only) and prochloraz-copper chloride-tebuconazole suspension were used as negative and chemical controls, respectively. Vertical bars indicate standard deviation of the means. Bars with the same letter do not differ significantly at *P* = 0.05. The experiment consisted of three replicates of 100 seeds each and the entire experiment was repeated thrice.

The optimum cell concentration of biocontrol agent is critical to obtain the expected control efficacy and apply it economically on a large scale. To determine the most effective and secure bacterial cell concentration for the control of BSR, we treated infected rice seeds with different concentrations of JBRS159. The BSR disease was significantly reduced to 46.7%, 36.7%, and 23.3% after treatment with 1 × 10^6^, 10^7^, and 10^8^ cfu mL^-1^ cells of JBRS159, respectively, compared to pathogen only challenged plants (58.0%) (Fig 1). Treatment with prochloraz-copper chloride-tebuconazole suspension reduced the disease to 8.0%. These results indicated that 1 × 10^8^ cfu mL^-1^ of JBRS159 could be used effectively for the suppression of BSR.

### Control effects of Si compounds on BSR disease

**Fig 2:**
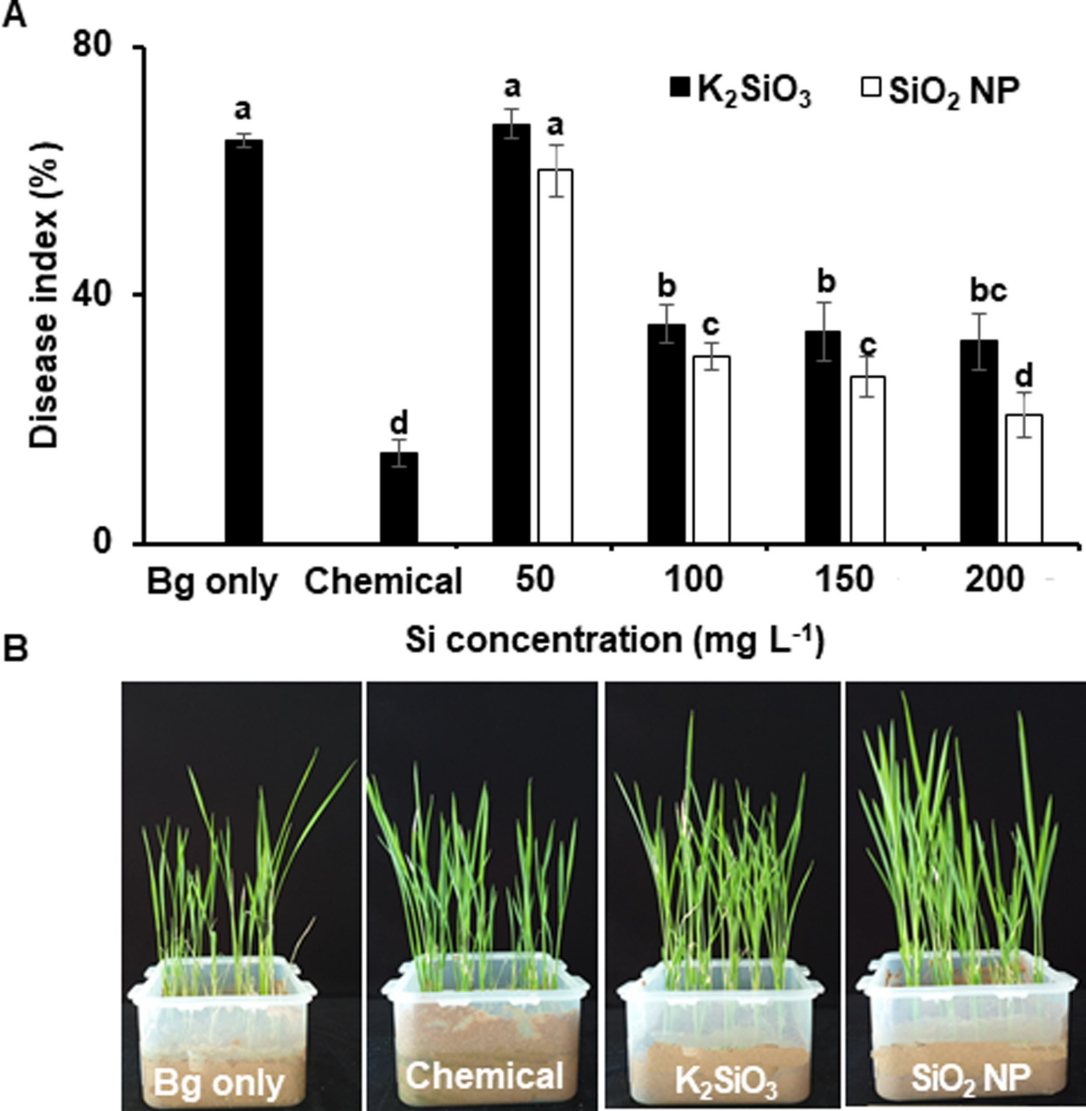
Control efficacy of various concentrations of each silicon compound on the bacterial seedling rot in rice plants. (A) Surface-sterilized rice seeds were challenge inoculated with *Burkholderia gluma*e, air-dried, and soaked in suspensions of K_2_SiO_3_ or SiO_2_ nanoparticles (NP) supplemented with 0.2% carboxymethyl cellulose (CMC). The treated seeds were sown in rice nursery bed soil. The seeds treated with sterile distilled water (DW) (Bg only) and prochloraz-copper chloride-tricyclazole suspension served as control and chemical treatments, respectively. Data are presented as mean ± standard deviation. Bars with the same letter do not differ significantly at *P* = 0.05. (B) The photos were taken 21 days after treatment.

Various concentrations of individual K_2_SiO_3_ and SiO_2_ nanoparticle were assayed for their control effects against BSR incidence to determine the optimum concentration of each Si compound. At 50 mg L^-1^ of K_2_SiO_3_ and SiO_2_ nanoparticle treatment, the disease incidence and progression were similar to those in plants treated with *B*. *glumae* only (Fig 2A and 2B). The disease was significantly suppressed at 100 mg L^-1^ of K_2_SiO_3_ (30.2%) and SiO_2_ nanoparticle (35.3%) compared to that in the pathogen only challenged plants (65.0%) (Fig 2A). In addition, we observed that the growth of rice plants treated with 100 mg L^-1^ SiO_2_ nanoparticle increased compared to that of the untreated control (Fig 2B). The BSR control efficacy was similarly maintained up to 400 mg L^-1^ for each Si compound. However, the growth was slightly retarded at concentrations ≥ 200 mg L^-1^. Therefore, the results indicated that 100 mg L^-1^ was the optimum concentration of K_2_SiO_3_ and SiO_2_ nanoparticle for further studies.

### Control efficacy of a mixture of JBRS159 and Si compounds

**Fig 3:**
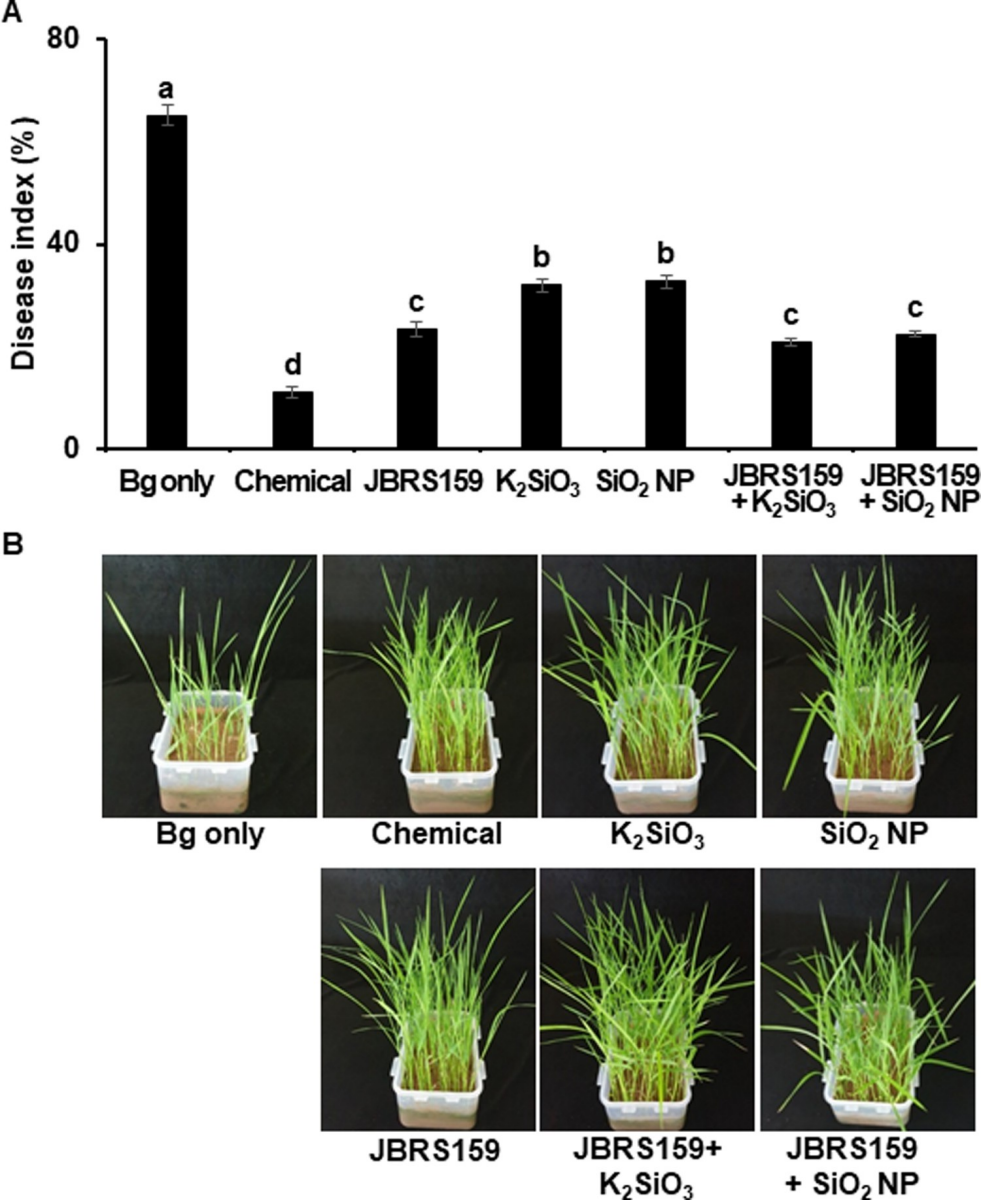
Control efficacy of bacterial seedling rot by treatment with *Cytobacillus firmus* JBRS159 and each silicon compound. (A) Surface-sterilized rice seeds were challenge inoculated with *Burkholderia gluma*e, air-dried, and soaked in suspensions of JBRS159, K_2_SiO_3_ and SiO_2_ nanoparticles (100 mg L^-1^), and a combination of JBRS159 and each silicon compound. Prochloraz-copper chloride-tricyclazole suspension was used as the chemical control. Data are presented as the mean ± standard deviation. Bars with the same letter do not differ significantly at *P* = 0.05. (B) The photos were taken 21 days after treatment.

The biocontrol efficacy of the mixture of optimum concentrations of JBRS159 (1 × 10^8^ cfu mL^-1^) and each Si compound (100 mg L^-1^) against BSR was compared with that of the individual treatments (Fig 3A and 3B). The disease indices in the pathogen only- and chemically treated pots were 65.3% and 11.3%, respectively. The disease was reduced to 23.5%, 32.0%, and 32.8% after treatment with JBRS159, K_2_SiO_3_, and SiO_2_ nanoparticles, respectively. The combination of JBRS159 with K_2_SiO_3_ or SiO_2_ nanoparticles further reduced the disease to 21.0% and 22.5%, respectively, compared to that with the individual treatments. The results indicated that simultaneous treatment of JBRS159 cells with Si improved their biocontrol efficacy compared to individual treatments with either Si compound, whereas the control efficacy of the mixture was not significantly increased compared to treatment with JBRS159 alone.

### Growth promotion of Arabidopsis and rice plant by JBRS159 and Si compounds

**Fig 4:**
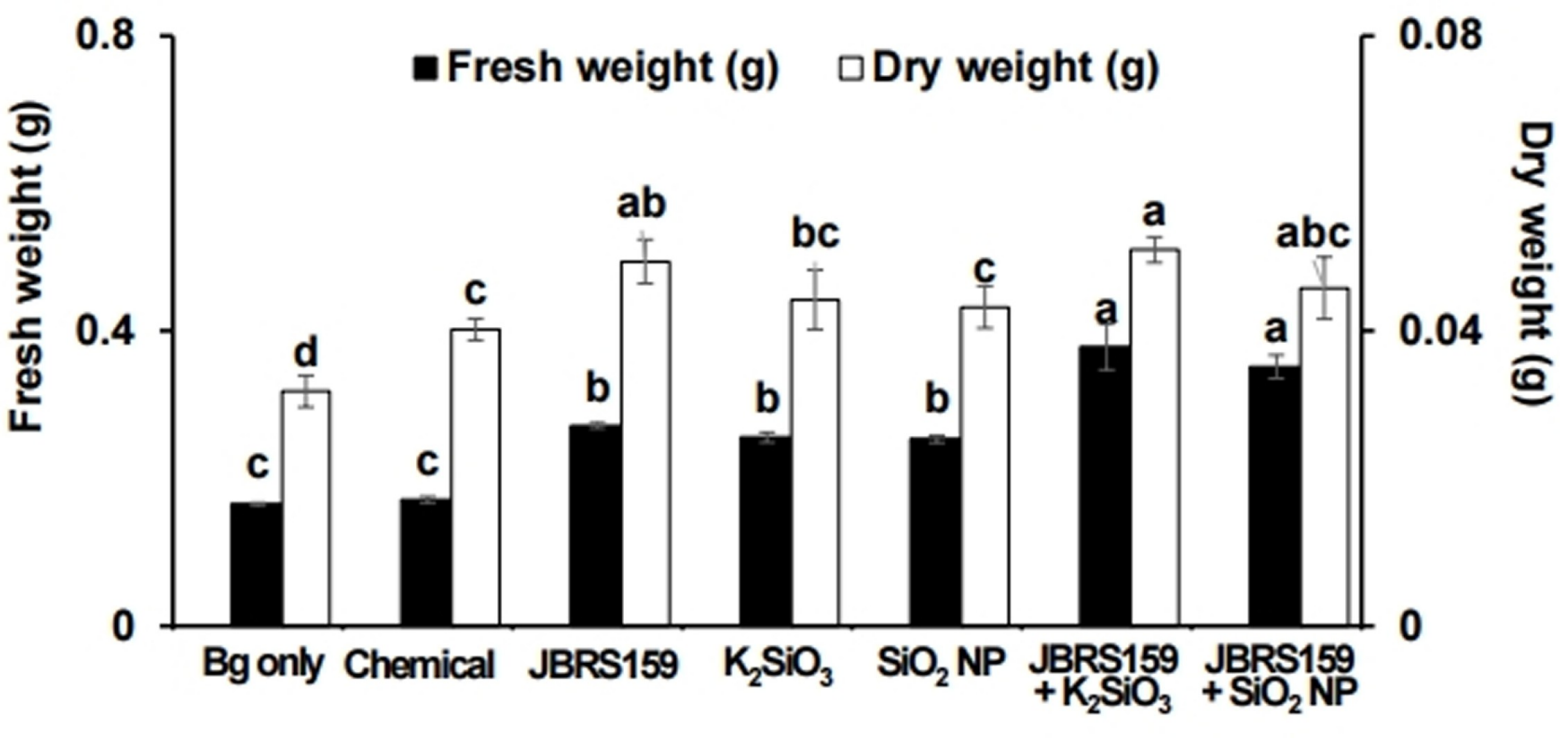
Effect of *Cytobacillus firmus* JBRS159 and silicon compounds on growth of rice plant. Surface-sterilized rice seeds were challenge inoculated with *Burkholderia gluma*e, air-dried, and soaked in *C*. *firmus* JBRS159 (JBRS159) bacterial suspension, K_2_SiO_3_, and SiO_2_ nanoparticles (100 mg L^-1^), and a combination of JBRS159 with each silicon compound. Data are presented as the mean ± standard deviation. Bars with the same letters do not differ significantly at *P* = 0.05.

Many crops such as rice and wheat take up Si, resulting in resistance to pathogen penetration and lodging. In the present study, we assessed the effect of each Si compound on the growth of *Arabidopsis* and rice plants. The fresh weight of *Arabidopsis* was significantly increased by treatment with JBRS159, K_2_SiO_3_, or SiO_2_ nanoparticles (100 mg L^-1^) compared with the untreated control (S2 and S3 Figs). The fresh weight increased by 45.8% and 21.1% with the combined treatment of JBRS159 with K_2_SiO_3_ or SiO_2_ nanoparticles, respectively, compared to the control, whereas the increase was not significant compared to the treatments with JBRS159 or individual Si compounds. The number of lateral roots was also significantly increased following treatment with JBRS159 or the Si compound. The combination of JBRS159 and K_2_SiO_3_ increased the number of lateral roots to approximately 86.8% compared to that of the control and the root architecture was observed to become dense and healthy (S3 Fig). Application of JBRS159, K_2_SiO_3_, and SiO_2_ nanoparticles also increased the fresh and dry weights of rice compared with those of the untreated control (Fig 4). The combination of JBRS159 with K_2_SiO_3_ or SiO_2_ increased fresh weight of rice plants by 39.4% and 29.4%, respectively, compared with that of JBRS159 alone, whereas dry weight was not significantly different between the combination and individual treatments. Rice plants treated with the combination of K_2_SiO_3_ and JBRS159 were healthier than those treated individually.

### Antagonism of JBRS159 and Si compounds against bacterial strains

The antagonistic activity of JBRS159 against bacterial pathogens of rice was investigated to understand the mode of action underlying the suppression of BSR. JBRS159 showed no antagonism against bacterial pathogens such as *B*. *glumae*, *B*. *plantari*, *B*. *gladioli*, *A*. *avenae*, and *X*. *o*. pv. *oryzae* (S4 Fig). These results indicated that the biocontrol activities of JBRS159 were not exerted by antagonistic activities.

K_2_SiO_3_ and SiO_2_ nanoparticle did not inhibit the growth of plant associated bacterial strains such as *B*. *glumae*, JBRS159, *B*. *velezensis*, *B*. *pyrrocinia*, and *P*. *parafulva* up to a concentration of 500 mg L^-1^ (S5 Fig). A concentration of 1000 mg L^-1^ slightly inhibited the growth of bacterial strains. These results indicate that the biocontrol activity of K_2_SiO_3_ or SiO_2_ nanoparticle (100 mg L^-1^) was not due to direct antibacterial activity against *B*. *glumae* and each Si compound is compatible with JBRS159, guaranteeing simultaneous application.

### Competition for nutrients between JBRS159 and *B*. *glumae*

**Fig 5:**
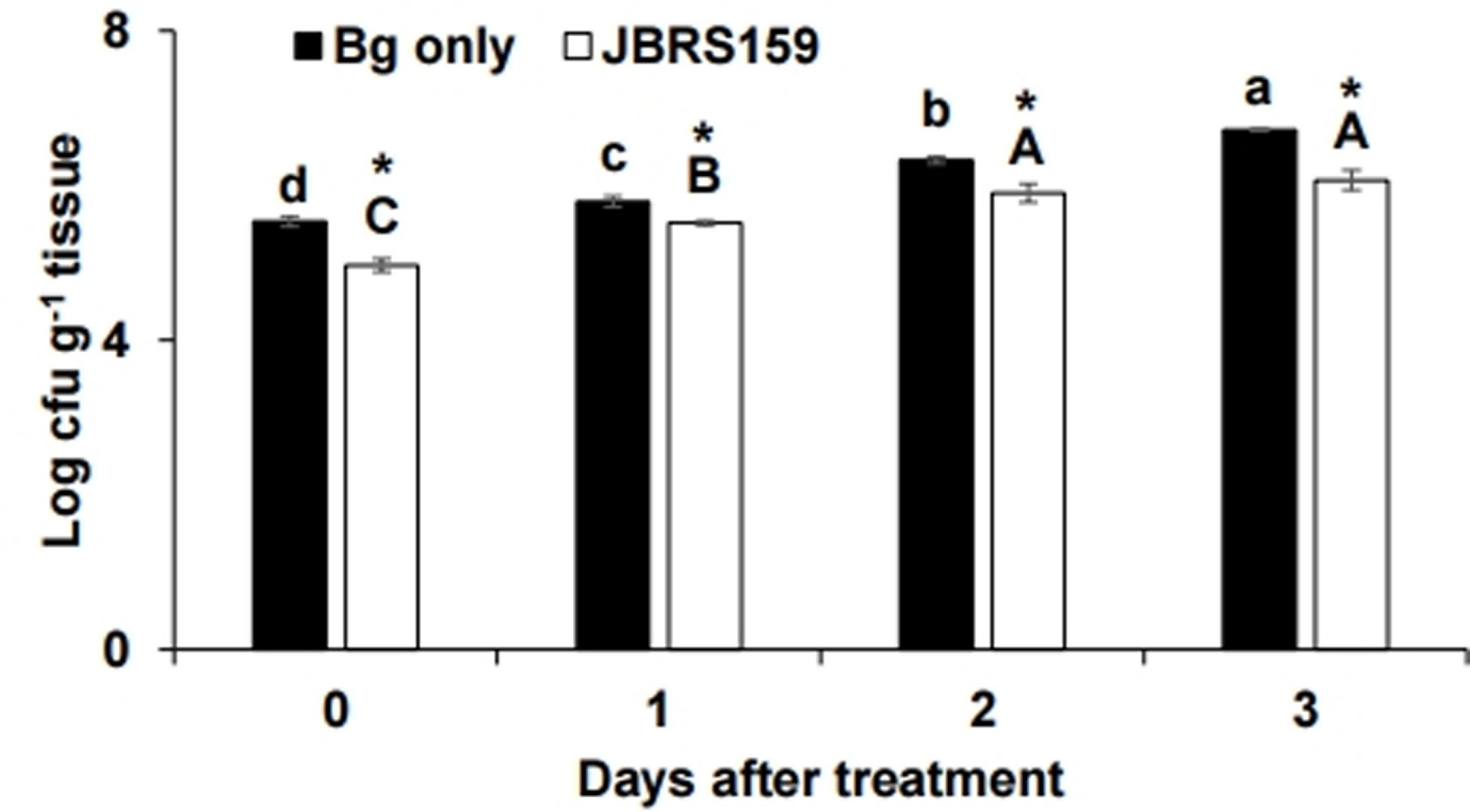
Population of *Burkholderia glumae* in rice. Surface-sterilized rice seeds were challenged with chloramphenicol-resistant *B*. *glumae* and treated with C*ytobacillus firmus* JBRS159. Populations of *B*. *glumae* in treated seeds and emerging radicles were analyzed using serial dilution plating method. Data are presented as the mean ± standard deviation. Bars with the same letters do not differ significantly at *P* = 0.05. Values marked by an asterisk (*) are significantly different at each time point at P < 0.05 according to Student’s *t*-test.

**Fig 6:**
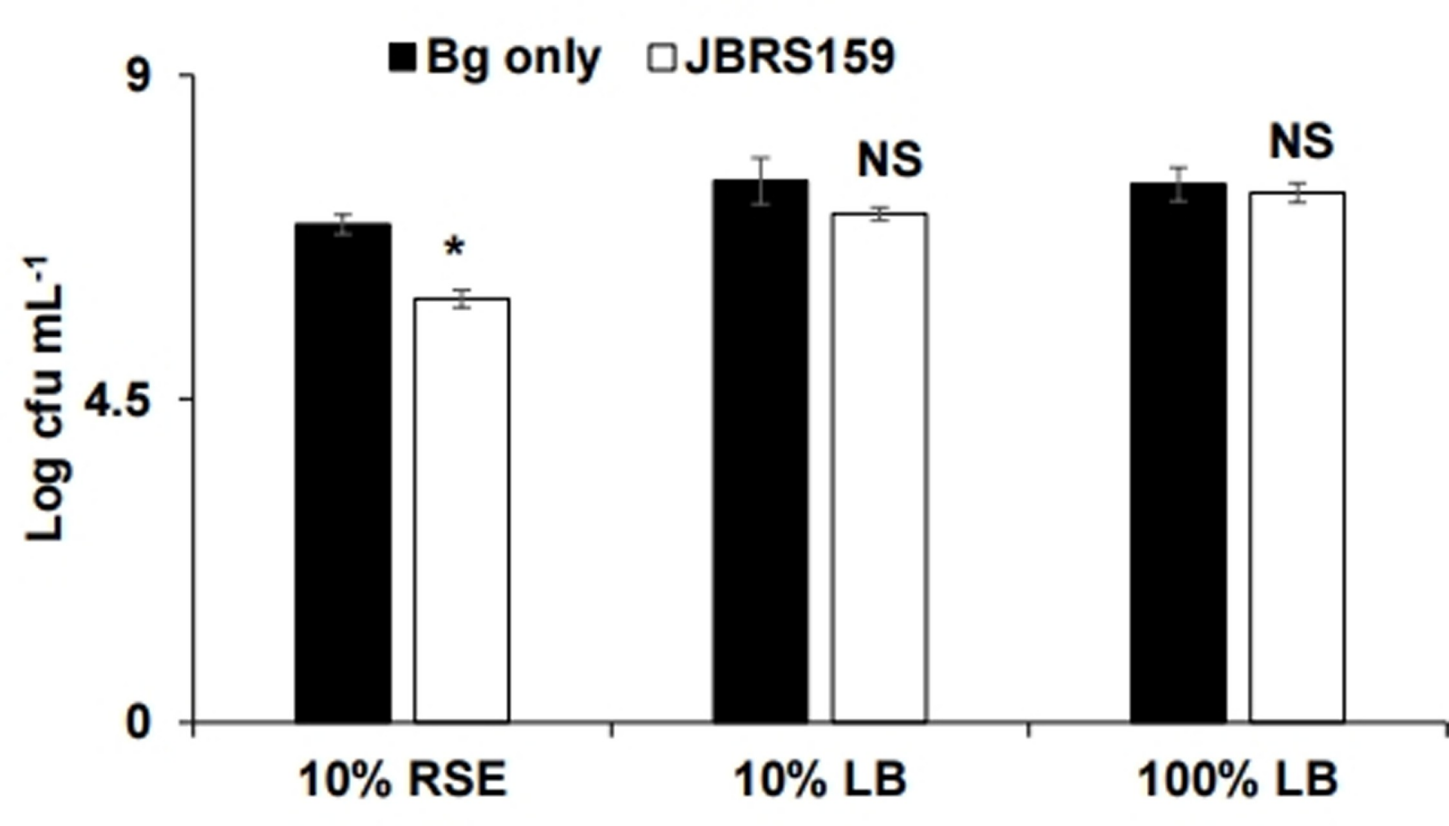
Growth of *Burkholderia glumae* in various nutrient conditions. *Burkholderia glumae* was incubated with *Cytobacillus firmus* JBRS159 in 10% rice seed exudate (RSE), 10% LB or 100% LB broth, and growth rates were recorded by serial dilution plating method. Data represent the mean ± standard deviation of the replications. Values marked by an asterisk (*) are significantly different for each media at P < 0.05 according to Student’s *t*-test.

To assess the effect of JBRS159 on the survival of *B*. *glumae* in the treated niches, the populations of the pathogen were estimated by serial dilution plating method. The populations of *B*. *glumae* in seeds and radicles were significantly reduced by the treatment of JBRS159 (Fig 5). The population of *B*. *glumae* in JBRS159 applied niches (1.2 × 10^6^ cfu g^-1^) was significantly suppressed compared to *B*. *glumae* only treatment (5.2 ×10^6^ cfu g^-1^) after 3 days of treatment. The results indicate that JBRS159 suppresses the survival of *B*. *glumae* in the rice seeds and roots. When *B*. *glumae* was cultured simultaneously with JBRS159 by separation with PTFE membrane, the growth in 10% RSE was significantly suppressed by the presence of JBRS159 (Fig 6). However, when they were grown together in 10% LB or full-strength LB medium, there was no significant difference in the growth of *B*. *glumae*. Taken together, the results suggest that JBRS159 suppressed the survival or establishment of *B*. *glumae* in the treated niches via nutrient competition.

### Production of growth and biocontrol stimulating compounds by JBRS159

The traits contributing to both activities were investigated to further understand the modes of action of the plant growth-promoting and disease-suppressing abilities of JBRS159. JBRS159 produced siderophores and proteases but not hydrogen cyanide (S6 Fig). JBRS159 solubilized phosphate, whereas Si was not mobilized, and produced IAA, but not cytokinin and gibberellic acid.

## Discussion

Bacterial panicle blight (BPB) is currently widespread in many rice-growing countries [1–3] and is considered a serious bacterial disease that is difficult to control in the field because infection-inducing outbreaks occur at the flowering stage and infected pathogens grow in the grain [5,8]. The use of infected seeds causes BSR in rice nurseries, and the bacterial population proliferates in plant tissues, causing epidemics in the field; the infected rice seeds are the primary source of inocula in the following season [7,46]. In this context, the disinfection of pathogen-contaminated seeds by coating them with microbes and eco-friendly materials can be an efficient practice for managing the incidence of BPB.

In the present study, we selected a potent biocontrol agent, *C*. *firmus* to control BSR. The strain JBRS159 exhibited more than 60% efficacy in the control of BSR and promoted an increase in fresh weight and lateral roots of *Arabidopsis* as well as growth in rice plants. The biocontrol of BSR by potential bacterial strains, including *Bacillus* has been reported in many studies [10,12,47]. For example, *C*. *firmus* (formerly known as *Bacillus firmus*) has been reported to promote plant growth and antagonistic activity against nematodes [48–50]. Recent studies have indicated that *C*. *firmus* produces IAA, and the antimicrobial activities of silver nanoparticles synthesized using *C*. *firmus* make the strain of potential interest [51,52]. In our study, JBRS159 did not show direct antagonism against bacterial and fungal pathogens but produced siderophores and showed protease activity. The JBRS159 genome (NCBI Acc. No. JAQZDS000000000) contained genes related to the biosynthesis and production of siderophores, hydrogen cyanide, acetoin, and IAA [32], which corresponded to the bioassay results of this study.

The population of *B*. *glumae* in rice seeds applied with JBRS159 was significantly decreased compared to pathogen-only treated seeds, which indicates the suppression of survival and establishment of *B*. *glumae* by JBRS159. In addition, the growth of *B*. *glumae* in RSE was significantly decreased by the presence of JBRS159, while there was no suppression of growth in 10% LB or 100% LB, which indicates nutrient absorption by JBRS159 in nutrient-poor conditions restricts the growth of *B*. *glumae*. Competition for nutrients and niches was reported as an indirect antagonistic mechanism in plant growth-promoting bacteria [53]. Recently, *Bacillus velezensis* reduced *B*. *glumae* in rice plants by competing for the niche, which consequently reduced disease symptoms [12]. *B*. *glumae* in rice seeds can colonize seedlings and gradually establish a steady population over time, and then can survive endophytically utilizing rice plants as a habitat to eventually form panicle blight symptoms [54]. The reduction of *B*. *glumae* in seeds and developing seedlings through competition for niches and nutrients can suppress the incidence of seed-borne BPB. Overall, the results of this study indicate that JBRS159 suppressed *B*. *glumae* by depriving nutrients, which consequently limits survival and establishment of the pathogen in plants. The specific nutrient component for the biocontrol activity of JBRS159 needs further study.

Many crops such as rice, wheat, maize, and potatoes have been known to accumulate high quantities of Si. Absorbed Si increases abiotic and biotic stress tolerance and enhances fertilizer use efficiency, which results in increased crop growth and yield [19,35,55,56]. SiO_2_ nanoparticles and soluble Si(OH)_4_ induce systemic acquired resistance in *Arabidopsis*, which involves the salicylic acid pathway [26]. Our study further established that soluble K_2_SiO_3_ or SiO_2_ nanoparticles are effective in the biocontrol of BSR, either individually or in combination with JBRS159. The co-application of biocontrol agents and Si has been reported to increase the efficacy of biocontrol activity. The addition of soluble K_2_SiO_2_ to *Serratia marcescens* and *Trichothecium roseum* increased the Si content in zucchini leaves, which suppressed the incidence of powdery mildew [57]. The toxicity of Ag nanoparticles was reduced by the treatment of *Bacillus thuringiensis* KVS2 with Si, promoting the growth of Indian mustard [18]. Co-inoculation with *Enterobacter* sp. UPMSSB7 and arbuscular mycorrhizal fungi (*Glomus mosseae*) in combination with Si significantly reduced the incidence of white root rot in rubber plants [58]. In the present study, the combination of JBRS159 with K_2_SiO_3_ or SiO_2_ nanoparticles reduced the disease of BSR more effectively than treatment with either K_2_SiO_3_ or SiO_2_ nanoparticles alone, but this was not significantly different from the JBRS159 only treatment.

Silicon has been reported to positively influence root development and diameter, main root length, and root biomass of soybeans grown under adverse environmental conditions [59]. Our results also indicated a positive influence of Si on *Arabidopsis* root growth. The dry weight, root volume, chlorophyll content of rubber plants, and Si content in the roots and shoots of plants were significantly increased by the co-inoculation of *Enterobacter* sp. and *G*. *mosseae* with Si [58]. In the present study, the fresh weights of *Arabidopsis* and rice plants were increased by combined treatment with JBRS159 and each Si compound compared to that with individual treatments with K_2_SiO_3_ or SiO_2_ nanoparticles or JBRS159. The combination of JBRS159 and K_2_SiO_3_ altered the morphology of *Arabidopsis* roots to a greater extent than the control, which enhanced the overall growth of the plants. However, the biocontrol and plant growth-promoting capacities did not increase as much as their sum. Because there was no direct antagonism against bacterial or fungal pathogens by either form of Si at a concentration of 100 mg L^-1^, direct inhibition of the growth of pathogens is not a mode of action to suppress the incidence of BSR. JBRS159 produces siderophores and IAA and solubilizes phosphate, which is beneficial for plant growth [5,8,45]. Microorganisms play an essential role in solubilizing Si from unavailable forms of silicates, enhancing plant growth and defense mechanisms, and increasing soil fertility [17,60,61]. In the present study, JBRS159 did not mobilize Si. Our results indicated that supplementation with Si compounds increased plant growth and disease control activities, presumably by complementing the capacity of JBRS159. The differences in the activities of K_2_SiO_3_ and SiO_2_ nanoparticles in disease suppression and plant growth promotion require further investigation. In addition, the mechanisms underlying the reinforcement of biocontrol and growth-promoting activities by combined treatment with Si and JBRS159 warrant further study.

SiO_2_ nanoparticles are absorbed through the stomata of *Arabidopsis* and induce disease resistance by remaining in the extracellular air spaces of leaves, which are safe for plants and the environment. However, high Si(OH)_4_ concentrations cause stress in *Arabidopsis* [26]. Silver nanoparticles (550 mg kg^-1^) exhibited a positive effect on species richness by increasing the population of diazotrophic bacteria, such as *Bradyrhizobium*, *Nitrospira*, and *Nitrosovibrio* without any toxic effects on microbes [62]. A 25 μM concentration of Si in sodium silicate promoted growth of *Brassica juncea* seedlings [18]. In the present study, K_2_SiO_3_ or SiO_2_ nanoparticles did not inhibit the growth of bacterial and fungal pathogens or JBRS159 at concentrations up to 500 mg L^-1^. A concentration of 1000 mg L^-1^ inhibited the growth of bacterial pathogens and JBRS159. These results indicate that JBRS159 is compatible with K_2_SiO_3_ or SiO_2_ nanoparticle at 100 mg L^-1^ and can be combined to increase and secure control efficacy.

In conclusion, eco-friendly methods for BSR control using the biocontrol agent JBRS159 or Si compounds (K_2_SiO_3_ or SiO_2_ nanoparticle) were achieved. JBRS159, K_2_SiO_3_, and SiO_2_ nanoparticle independently suppressed the BSR disease and promoted plant growth. The interactive effect between JBRS159 and Si compounds was not as high as the sum of the two but complementary to the capacity. JBRS159 might suppress disease by depriving nutrients from the pathogen. Overall, JBRS159 and K_2_SiO_3_ or SiO_2_ nanoparticle are efficient and safe alternatives for BSR control and growth promotion. The efficacy of JBRS159 and Si compounds in the control of BSR and BPB in the field requires further study.

## Supporting information

S1 Fig Disease symptoms used for disease indices of this study. Rice seeds challenged with *Burkholderia glumae* were estimated for disease severities using disease index scales 0–4: 0 = seedlings with no symptoms and vigorous as control, 1 = seedlings with pale yellow leaves, 2 = seedlings with severe chlorosis and stunting, 3 = seedlings with complete discoloration and rotting, and 4 = seeds completely rotted without development.
(TIF)

S2 Fig Effect of JBRS159 and silicon compounds on the growth of *Arabidopsis*. Seeds of *Arabidopsis thaliana* Col-0 were treated with suspensions of JBRS159, and K_2_SiO_3_ and SiO_2_ nanoparticles (100 mg L^-1^), and a combination of JBRS159 and each silicon compound. The treated seeds were placed on half-strength MS medium, and data were recorded 14 d after growth. Data are presented as the mean ± standard deviation. Bars with the same letters do not differ significantly at *P* = 0.05.
(TIF)

S3 Fig Effect of *Cytobacillus firmus* JBRS159 and silicate on the growth of *Arabidopsis*. Seeds of *Arabidopsis thaliana* Col-0 were treated with suspensions of *C*. *firmus* JBRS159 (JBRS159), K_2_SiO_3_, and SiO_2_ nanoparticles (100 mg L^-1^), and a combination of JBRS159 and each silicon compound. The treated seeds were placed on half-strength Murashige and Skoog (MS) medium. Photos were taken 14 d after incubation and a close-up view of root architecture is shown below each plate.
(TIF)

S4 Fig Antagonism of *Cytobacillus firmus* JBRS159 against bacterial plant pathogens. Antagonism against bacterial pathogens, (A) *Burkholderia glumae*, (B) *Burkholderia gladioli*, (C) *Burkholderia plantarii*, (D) *Xanthomonas oryzae pv*. *oryzae*, and (E) *Acidovorax avenae* was tested using a dual inoculation technique.
(TIF)

S5 Fig Effect of potassium silicate and silica nanoparticles on bacteria. The antibacterial activity of K_2_SiO_3_ or SiO_2_ nanoparticles was assessed using overlay inoculation. K_2_SiO_3_ or SiO_2_ nanoparticles (20 µL) was dropped on paper disks placed on the media mixed with each bacterial cell, (A) *Burkholderia glumae*, and (B) *Cytobacillus firmus* JBRS159. Each paper disc contained 20 µL of each silicate concentration; clockwise from top 100, 200, 500, and 1000 mg L^-1^. The paper disk in the center is the control. Photos were taken 2 d after incubation at 28°C.
(TIF)

S6 Fig Plant growth-promoting and disease-suppressing activities of *Cytobacillus firmus* JBRS159. (A) Siderophore production was assessed by a change in the color of chrome azurol S (CAS) medium from blue to orange, (B) phosphate solubilization was determined using NBRIP medium by induction of a clear zone around the colonies, (C) Silicate solubilization was determined using glucose agar medium, (D) Protease activity was determined using casein as the substrate.
(TIF)
