# Supplementary figures and images for: Biocontrol of bacterial seedling rot of rice plants using combination of Cytobacillus firmus JBRS159 and silicon
Source: PLoS One. 2023 Aug 14;18(8):e0290049. doi: 10.1371/journal.pone.0290049 (PMC10424872; doi:10.1371/journal.pone.0290049)

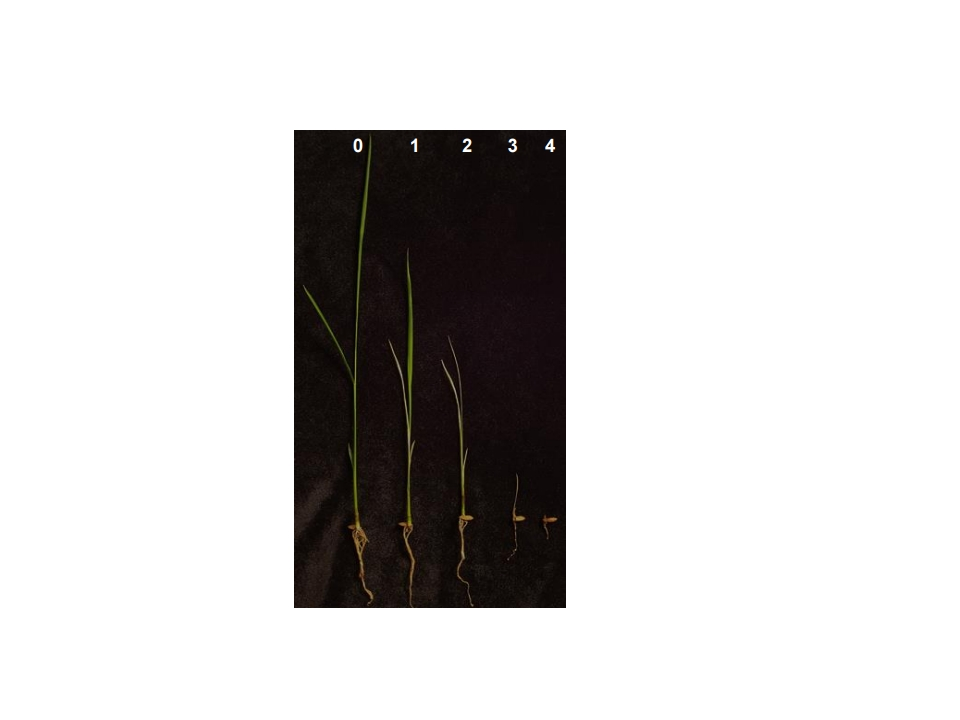

Supplement: S1 Fig — Rice seeds challenged with Burkholderia glumae were estimated for disease severities using disease index scales 0–4: 0 = seedlings with no symptoms and vigorous as control, 1 = seedlings with pale yellow leaves, 2 = seedlings with severe chlorosis and stunting, 3 = seedlings with complete discoloration and rotting, and 4 = seeds completely rotted without development. (TIF) [file pone.0290049.s001.tif]

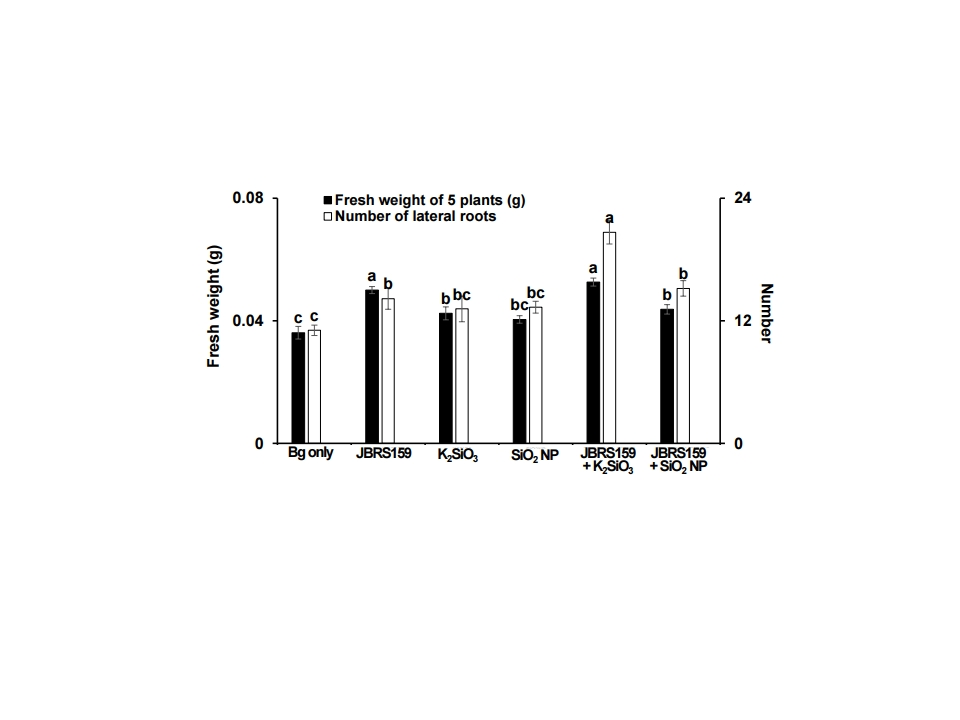

Supplement: S2 Fig — Seeds of Arabidopsis thaliana Col-0 were treated with suspensions of JBRS159, and K2SiO3 and SiO2 nanoparticles (100 mg L-1), and a combination of JBRS159 and each silicon compound. The treated seeds were placed on half-strength MS medium, and data were recorded 14 d after growth. Data are presented as the mean ± standard deviation. Bars with the same letters do not differ significantly at P = 0.05. (TIF) [file pone.0290049.s002.tif]

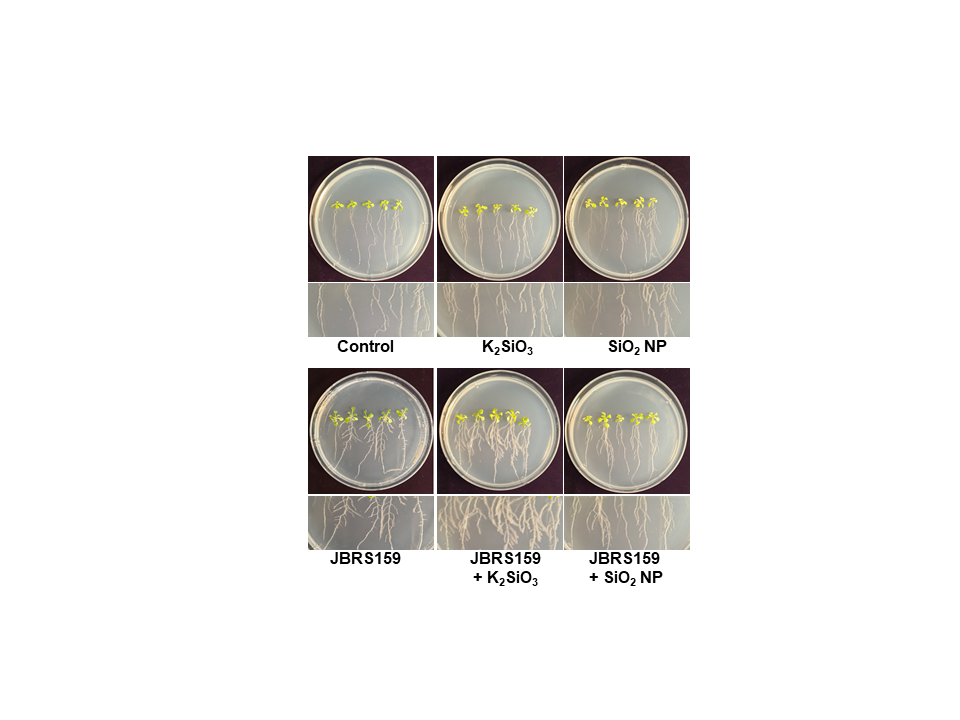

Supplement: S3 Fig — Seeds of Arabidopsis thaliana Col-0 were treated with suspensions of C. firmus JBRS159 (JBRS159), K2SiO3, and SiO2 nanoparticles (100 mg L-1), and a combination of JBRS159 and each silicon compound. The treated seeds were placed on half-strength Murashige and Skoog (MS) medium. Photos were taken 14 d after incubation and a close-up view of root architecture is shown below each plate. (TIF) [file pone.0290049.s003.tif]

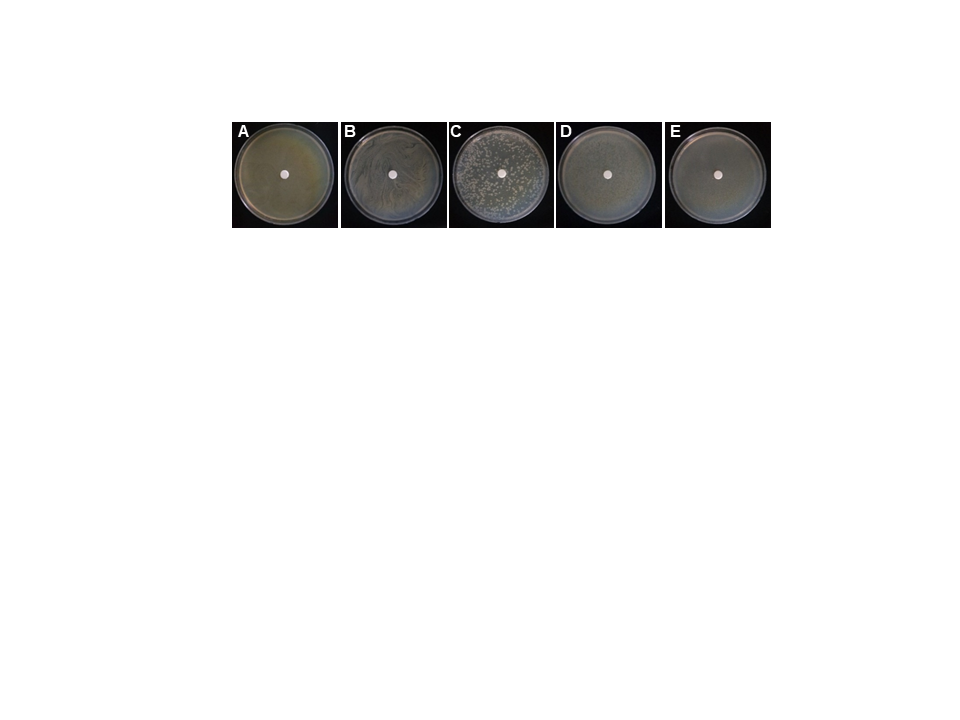

Supplement: S4 Fig — Antagonism against bacterial pathogens, (A) Burkholderia glumae, (B) Burkholderia gladioli, (C) Burkholderia plantarii, (D) Xanthomonas oryzae pv. oryzae, and (E) Acidovorax avenae was tested using a dual inoculation technique. (TIF) [file pone.0290049.s004.tif]

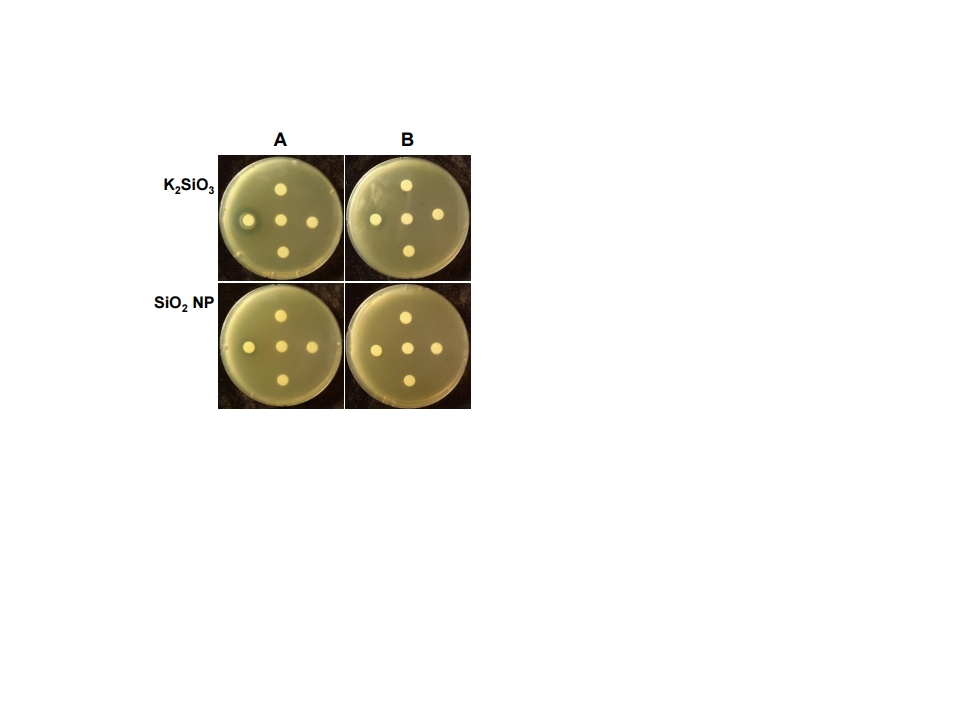

Supplement: S5 Fig — The antibacterial activity of K2SiO3 or SiO2 nanoparticles was assessed using overlay inoculation. K2SiO3 or SiO2 nanoparticles (20 µL) was dropped on paper disks placed on the media mixed with each bacterial cell, (A) Burkholderia glumae, and (B) Cytobacillus firmus JBRS159. Each paper disc contained 20 µL of each silicate concentration; clockwise from top 100, 200, 500, and 1000 mg L-1. The paper disk in the center is the control. Photos were taken 2 d after incubation at 28°C. (TIF) [file pone.0290049.s005.tif]

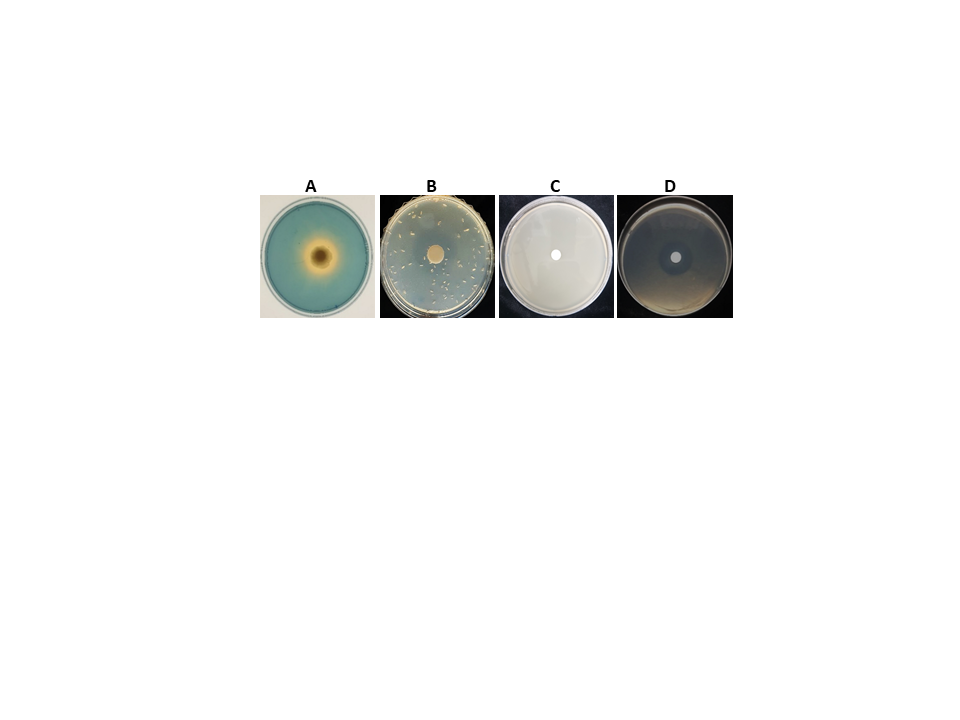

Supplement: S6 Fig — (A) Siderophore production was assessed by a change in the color of chrome azurol S (CAS) medium from blue to orange, (B) phosphate solubilization was determined using NBRIP medium by induction of a clear zone around the colonies, (C) Silicate solubilization was determined using glucose agar medium, (D) Protease activity was determined using casein as the substrate. (TIF) [file pone.0290049.s006.tif]
